# Biophysical Investigations Elucidating the Mechanisms of Action of Antimicrobial Peptides and Their Synergism

**DOI:** 10.3390/biom8020018

**Published:** 2018-04-18

**Authors:** Arnaud Marquette, Burkhard Bechinger

**Affiliations:** Université de Strasbourg/CNRS, UMR7177, Institut de Chimie, 4, rue Blaise Pascal, 67070 Strasbourg, France; marquette@unistra.fr

**Keywords:** magainin, cecropin, membrane topology, local disorder, membrane pore, membrane macroscopic phase, soft membrane adapt and respond, also transiently (SMART) model, carpet model, toroidal pore, peptide-lipid interactions

## Abstract

Biophysical and structural investigations are presented with a focus on the membrane lipid interactions of cationic linear antibiotic peptides such as magainin, PGLa, LL37, and melittin. Observations made with these peptides are distinct as seen from data obtained with the hydrophobic peptide alamethicin. The cationic amphipathic peptides predominantly adopt membrane alignments parallel to the bilayer surface; thus the distribution of polar and non-polar side chains of the amphipathic helices mirror the environmental changes at the membrane interface. Such a membrane partitioning of an amphipathic helix has been shown to cause considerable disruptions in the lipid packing arrangements, transient openings at low peptide concentration, and membrane disintegration at higher peptide-to-lipid ratios. The manifold supramolecular arrangements adopted by lipids and peptides are represented by the ‘soft membranes adapt and respond, also transiently’ (SMART) model. Whereas molecular dynamics simulations provide atomistic views on lipid membranes in the presence of antimicrobial peptides, the biophysical investigations reveal interesting details on a molecular and supramolecular level, and recent microscopic imaging experiments delineate interesting sequences of events when bacterial cells are exposed to such peptides. Finally, biophysical studies that aim to reveal the mechanisms of synergistic interactions of magainin 2 and PGLa are presented, including unpublished isothermal titration calorimetry (ITC), circular dichroism (CD) and dynamic light scattering (DLS) measurements that suggest that the peptides are involved in liposome agglutination by mediating intermembrane interactions. A number of structural events are presented in schematic models that relate to the antimicrobial and synergistic mechanism of amphipathic peptides when they are aligned parallel to the membrane surface.

## 1. Introduction

Antimicrobial peptides (AMPs) are effectors of the innate immune system which provide a first line of defense against a multitude of pathogenic microorganisms. Higher organisms release AMPs immediately when infections by bacteria or fungi occur [1,2]. They have been found in a wide variety of species from the plant and animal kingdom, including humans [3]. Furthermore, many peptides produced by microorganisms have been identified and investigated [4,5]. After their first discovery decades ago [6,7,8], many new sequences have been added to the corresponding data bases [9,10] and they have been investigated by a wide variety of techniques to better understand their mechanisms of action. Since the worldwide re-emergence of infectious diseases and a rapid increase in multi-resistant pathogens [11] they bear great promise to lead the way to new classes of antibiotics capable of counteracting the continuously increasing threat by resistant microorganisms; thus the declining number of effective pharmaceutical agents can be complemented. Whereas many natural peptides have potential topical applications, they are considered unsuitable for oral intake because of their fast degradation by proteases. However, peptides can be modified and made unavailable during transport by incorporation into nanostructures or by fixation to surfaces [12,13,14], thus they are protected and able to reach their target. Alternatively, understanding the mechanism of action of the natural sequences paves the way to designing molecules with favorable properties that mirror the essential characteristics of the template compounds. Therefore, the study of antimicrobial peptides (AMPs), which have evaded bacterial resistance during millions of years of evolution [2], promises to reveal novel strategies for the development of new lines of antibiotics.

The physico-chemical characteristics of antimicrobial peptides discussed in this paper in combination with a multitude of investigations indicates that they interact with lipid bilayers and interfere with the barrier function of bacterial membranes. In contrast, molecules that specifically target proteinaceous receptors can be made inefficient by mutagenesis of one or a few sites, and it is much less likely that bacteria develop resistance to compounds whose primary target is the destruction of the physico-chemical properties of the lipid membrane [15]. Membrane-active peptides exhibit a wide range of structural features some being helical in their bilayer-associated state [16,17], others forming cyclic [18,19,20,21] and/or β-sheet arrangements [22,23,24,25,26]. Indeed, following the insights gained from the studies of cationic amphipathic antimicrobial peptides a number of small amphipathic molecules [27,28], pseudopeptides [21,29,30,31,32,33,34,35,36], and polymers [37] have been designed and investigated, and found to also exhibit potent antimicrobial activities.

Here, some of the underlying research efforts shall be presented that have led to important mechanistic insights and the design of new compounds. The review focuses on linear cationic peptides such as magainins [38], cecropins and designed peptides [39,40,41] (amino acid sequences are provided in Table 1), antimicrobial peptides which have been described and investigated early on. In the following, insights with these AMPs provided guidelines for the design of new compounds and initiated the search for, and investigation of, related sequences. Despite decades of research, new structural and dynamic features of membrane-associated AMPs are continuously being discovered [26,42] because these peptides can adopt a large diversity of conformations and topologies whose exchanges and interactions are governed by multiple equilibria [43]. Finally, the synergistic interactions between PGLa and magainins will be discussed because the covalent or non-covalent combination of compounds provides an alternative strategy to enhance their efficiency and further reduces their susceptibility to bacterial resistance.

Peptides from frogs and insects have already been described in the 1960s [7,44], while magainins and cecropins, which act as specific antimicrobial compounds, have been discovered with some delay [2,45]. These sequences are linear, highly cationic, and form amphipathic helices when interacting with membranes. They are thought to specifically disrupt the integrity of bacterial and fungal membranes by insertion, thereby inhibiting the growth of microorganisms and/or enter into the cell interior [46]. Thereby, they constitute a first line of defense when infections occur [2,45]. By also modulating the immune response of the host organisms, their efficiency is considerably enhanced, and because of this extension of functionalities, it has been suggested to rename them as ‘host defense peptides’ [47,48,49,50]. Indeed, metabolomics studies reveal complex reactions by the bacterial cells when exposed to AMPs, many being unique to a specific sequence [51,52].

Magainins and derivatives thereof have been extensively studied by biophysical approaches (e.g., [53,54,55,56,57]), with sometimes unexpected results, and the insights thus obtained have formed the basis for suggesting novel mechanisms of action of these peptides [43,58]. When added to membranes, magainins and cecropins were found to exhibit lytic activities. In some electrophysiological experiments, they also showed discrete multi-level conductivities [59,60,61,62], which in analogy to models that had been proposed for the hydrophobic alamethicin peptide or for large helical channel proteins, was taken as an indicator for transmembrane helical bundle formation [63]. However, it is important to emphasize that unlike the alamethicin channels, those recorded in the presence of magainins or cecropins are less well defined, erratic, and characterized by large variations [59,60,61]. These pronounced differences in electrophysiological recordings reflect distinct physico-chemical properties of these sequences, such as number of charges, hydrophobicity, and hydrophobic moment (Table 1).

Furthermore, magainin pore formation was investigated on a macroscopic scale by following the kinetics of calcein release from individual giant unilamellar vesicles (GUV) made from defined mixture of membrane lipids, dioleoylphosphatidylcholine/dioleoylphosphatidylglycerol DOPC/DOPG at different molar ratios [64]. These experiments indicate that after addition of the peptide, it takes minutes before the release of fluorophores sets in. Once the pores have been established, the vesicles, which are several micrometers in size, empty within 30 s. Because the rate-limiting step is the formation of the pore, rather than diffusion through the pore, magainin pore formation in GUVs follows an all-or-nothing mechanism. This observation agrees with calcein release experiments from suspensions of large unilamellar vesicles made from palmitoyl-oleoyl-phosphatidylcholine (POPC) and palmitoyl-oleoyl-phosphatidylglycerol (POPG) lipid membranes [65]. Conversely, for magainin 2, an all-or-nothing mechanism dominates in the presence of 50 mol % phosphatidylglycerol (PG) a more graded release is observed when the PG content is reduced to 20 mol % [65]. These experiments showed no indications of peptide oligomerization in either state [65]. The subsequent fluorophore release is a two-stage process [66]. An initial fast release has been associated with magainin interacting with only the outside monolayer, which causes an unbalance. Equilibration of the peptide concentration between the outer and inner leaflets results in the transient formation of very large pores [66]. Thereafter, smaller pores assure a slower release of fluorophore, but even these persistent openings are large enough to allow for the passage of molecules with a hydrodynamic radius of 3 nm (equivalent to a globular protein of molecular weight (MW) > 20 kDa) [66].

**Figure 1:**
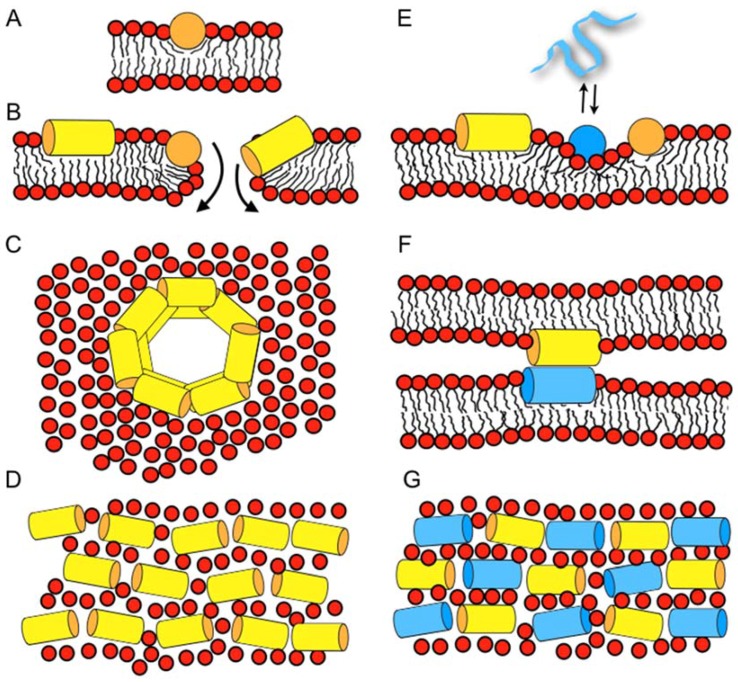
Schematic models illustrating how antimicrobial peptides work and interact with membranes (**A**–**D**), and how two peptides can interact synergistically in a membrane environment (**E**–**G**). (**A**) Peptides such as magainin partition into the membrane interface and cause disordering of the lipid packing. (**B**) Bilayer openings form stochastically when the peptide concentration increases locally, or when the membrane disrupts at high peptide-to-lipid ratios [72]. Along the openings, the peptides can insert and cross in in-planar or at tilted alignments. (**C**) In molecular dynamics calculations schematic, amphipathic helices have been simulated to form double belts [73], an arrangement which also agrees with the in-planar alignment of the peptide helices observed by solid-state nuclear magnetic resonance NMR spectroscopy [38]. (**D**) Fluorescence quenching experiments suggest mesophase structures formed by in-plane oriented helices [74]. (**E**) The membrane disruptive properties of one peptide (yellow) help the insertion of another one (blue), which by itself is less likely to partition into membranes of high negative curvature [75]. (**F**) Peptide-peptide contacts result in the agglutination of liposomes (Figure 3) [76], and could be responsible for synergistic enhancement of activities. (**G**) A more densely packed mesophase arrangement forms in the presence of two peptides with complementary charge distribution such as magainin 2 and PGLa [77]. Notably, multiple mechanisms, such as a combination of **E**, **F**, and **G** may apply. Panels **A**, **B**, **E**, and **F** show side views, panels **C**, **D**, and **G** show top views of the lipid bilayer.

Recently, microscopic imaging techniques were introduced into the field, which reveal the spatio-temporal binding of antimicrobial peptides to live bacteria and the related membrane permeabilization events. For example, the human peptide LL37 preferentially attacks septating *Escherichia coli* cells where the peptide is found associated with the septum and the curved regions of the outer membrane [67]. In non-septating cells, it prefers to bind to one of the endcaps. Influx of the AMPs to the periplasmic space results in cell shrinking, probably via an osmotic effect. After permeabilization of the outer membrane, there is a short delay before cytoplasmatic membrane permeabilization occurs. These openings of the outer and cytoplasmatic membranes are localized and persistent, rather than global and transient [68]. Notably, whereas many events observed on this cellular level resemble each other, the exact details vary with the antimicrobial compound when cationic polymers, longer or shorter peptides such as LL37, cecropin A, or melittin are compared to each other [69]. Furthermore, the events that happen with *E. coli* cells that are grown either under aerobic or anaerobic conditions have been compared to each other and correlated with mutagenesis experiments [70]. This data suggests that LL37 specifically affects the electron transport chain [70]. Notably, the permeabilization in the presence of alamethicin follows a different series of events, even though the data do not rule out a chaotic pore or a carpet mechanisms for this hydrophobic peptide [71]. Whereas a chaotic pore structure is shown in Figure 1A,B a peptide carpet is illustrated in reference [43].

Structural investigations show that the random coil structure of magainins in aqueous solution becomes helical once the peptide inserts into membrane environments [72]. This conformational transition has been identified to be a driving force of membrane association [78,79]. Importantly, both circular dichroism (CD) and solid-state nuclear magnetic resonance (NMR) spectroscopy on uniaxially oriented membranes indicate that the magainin helix is oriented parallel to the membrane surface, which results in membrane association being reversible [38]. The in-planar alignment has been confirmed for magainin 2 in membranes of different composition [38,54], for magainin analogues [80,81] and for a number of other linear cationic antimicrobial peptides [82,83,84,85,86]. In contrast, alamethicin with its much different characteristics in both electrophysiological recordings and physico-chemical properties (Table 1), has been found to form well-defined channels (reviewed e.g., in [17,87]) and to adopt stable transmembrane helical alignments in canonical dimyristoyl-phosphatidylcholine (DMPC) and (POPC) membranes [88,89,90,91,92]. However, even this peptide adopts in-plane alignments under certain conditions [42,93] emphasizing the dynamic nature of peptide-lipid interactions involving multiple equilibria [43]. CD spectroscopy has also been used to study the association kinetics of magainin to whole cells and lipopolysaccharides [94].

When investigated in more detail, the topology of magainin 2 has been found to be parallel to the membrane surface regardless of the lipid composition [38,54]. In contrast, a much wider range of alignments has been observed for its relative, PGLa [95,96] (Table 1). Interestingly, the difference becomes only apparent in membranes where both fatty acyl chains are saturated. For example, in DMPC, PGLa changes its tilt angle by up to 30 degrees upon an increase in peptide concentration [97]. The transition occurs at 0.5 to 2 mol % depending on the membrane hydration conditions [95,97]. A continuous range of tilt angles was observed when saturated phosphatidylcholine (PC) bilayers with decreasing hydrophobic thickness where investigated [95,96]. These studies reveal helical tilt angles that are suggestive of transmembrane orientations when thin phosphatidylcholine bilayers (C10 or C12 fatty acyl chains) are investigated [95]. When PGLa is studied in phospholipid bilayers carrying unsaturations (such as palmitoyl-oleoyl-phospholipids), the peptide remains stably aligned along the surface [38,95,98].

When an amphipathic peptide such as magainin resides in the bilayer interface, it pushes apart the lipids at the level of the head group and glycerol regions [54], which loosens the packing of the hydrophobic region (disordering effect, Figure 1A) [99,100]. The accompanying compensation by the membrane lipids results in a reduction of the membrane thickness [57,101]. Deuterium solid-state NMR measurements have indeed revealed a decrease of the order parameters in the bilayer interior upon addition of magainin 2, PGLa, and other amphipathic peptides [99,102,103,104]. Notably, the bilayer disruptive properties of such peptides have been estimated to cover a 50 Å radius [105,106].

The question arises for how in-plane oriented peptides can promote the passage of water, ions, and fluorescence dyes across the lipid bilayer. Molecular dynamics simulations have provided atomistic views on how this may be possible. They show not only the method by which hydrophobic peptides insert into the membrane to form peptide channels made from transmembrane helical arrangements [63], but also how in-plane oriented helices deform the lipid bilayer, and how their side chains reach to the opposite bilayer leaflet of the membrane, thus resulting in the formation of water-filled openings [73,107,108].

Lazaridis and co-workers report on 5–9 µs all-atom molecular dynamics simulations, starting from tetrameric transmembrane helical bundles of magainin or PGLa in 80–120 lipids of DMPC or DMPC/DMPG 3/1 [108]. During the simulations, the peptides lose their transmembrane orientation and adopt tilted configurations where magainin also occurs as an antiparallel dimer (illustrated in Figure 1B).

Furthermore, Vacha and coworkers present coarse-grained molecular dynamics (MD) of schematic amphipathic peptides and find evidence for a novel double belt arrangement where peptides oriented parallel to the bilayer plane form defined membrane openings (Figure 1C) [73]. The exact topology depends on the length of the peptide and its hydrophobicity distribution, but also on the membrane thickness [109]. All-atom 100 ns simulations in palmitoyl-oleoyl-phosphatidylethanolamine/ palmitoyl-oleoyl-phosphatidylglycerol (POPE/POPG) 3/1 of one, two, or eight peptides and 512 lipids show a stable in-plane topology of magainin and pleurocidin, some oligomerization, but no pore or supramolecular rearrangement within this time frame [110]. Finally, recent simulation work investigating magainin 2-lipopolysaccharide and ion interactions shall be mentioned [111].

Taken together, the molecular dynamics simulations provide a rather heterogeneous view of the magainin membrane interactions, where, despite some peptide-peptide interactions, pores form through stochastic rearrangements of peptides and lipids rather than through well-defined channel structures (Figure 1B). Although this view is in good agreement with electrophysiological recordings [59,60,61,62] the comparatively small size of the membrane patches and their relatively short duration does not yet capture the membrane lytic nature of the peptides, or the large pores that become apparent in dye release experiments [65,66].

Based on the GxxxG amino acid motif, which has been shown to be a dimer recognition sequence for transmembrane helical domains [112], a symmetric antiparallel dimer of PGLa has been assembled and then simulated by all-atom MD for up to 2 µs [113]. Although this time frame is too short to follow larger supramolecular rearrangements or the dissociation of preformed oligomers, the simulation provides interesting images of possible arrangements of PGLa in lipid bilayers. Unfortunately, to our knowledge so far, experimental proof for dimer formation in membrane environments such as solid-state NMR distance measurements is still missing, thus the exact reason for the change in topology when the peptide concentration increases (cf. ultra) [95,96,97] remains a matter of speculation. An interesting question in this context is the local environment of the GxxxG motif, which to our knowledge has only been shown to drive dimerization within the hydrophobic core of the membrane [112]. Dimerization thus assures that the polar backbone atoms of helical glycines are shielded from the hydrophobic surroundings. Along this line, dimer formation is suggestive of a deep membrane penetration of the PGLa helix, placing the two glycines in a non-polar environment.

The bilayer disruptive properties of amphipathic helices that are aligned along the membrane interface, with some of the helical cross-section interacting with the hydrophobic fatty acyl chain and the opposite face exposed to the polar head group, can be rationalized by the molecular shape concept that has been originally introduced to explain the phase behavior of lipids [114,115]. Geometrical considerations are used to explain why the cylindrical PC lipids arrange into extended bilayers, the cone shaped phosphatidylethanolamine (PE) have a tendency to adopt H_II_ phases and detergents with an inverted cone shape assemble into micelles. When compared to these lipids, surfactin, a cyclic peptide with a long fatty acyl chain [20], or the magainin 2 in-planar interfacial helix, use up much more space in the head group than the hydrophobic core region of the membrane, thereby resembling detergents (Figure 1A) [115]. A predictive model for the activities of linear cationic peptides, based on this and previous considerations [58,72,115], has recently been elaborated for magainin 2 and melittin [116].

The wide variety of observations made with magainins and other cationic amphipathic antimicrobial peptides has resulted in a number of seemingly contradictory models for their interactions and supramolecular arrangement in bacterial membranes. These include toroidal pores [117,118], the ‘carpet’ model where peptides cover the membrane surface at alignments parallel to the surface (Figure 1B) [119], or random aggregates within the membrane [120]. Furthermore, in electrophysiological recordings, channel-like events have been observed [59,60,61], whereas at high peptide concentration, the formation of worm like structures, disk-shaped particles, or micelles have been shown to occur [5,8,102,121]. A model should provide explanations for all of such a wide variety of features.

It is important to note that the peptides are flexible and highly dynamic, and can adjust their conformation and topology to the environment (e.g., [122,123]). In a related manner, lipid bilayers are soft, can change shape and thickness, and are capable of adjusting to the presence of peptides or to other environmental factors. To take into account the flexibility and dynamics of both the peptides and the lipid membrane, the SMART model has been introduced, where ‘Soft Membranes Adapt and Respond, also Transiently’, in the presence of antimicrobial peptides (or other external stimuli). As suggested by its name the model takes into account that lipid membranes can adapt to some extent to the disruptive properties of the peptides, but undergo macroscopic phase transitions at higher peptide concentrations, locally (Figure 1B) or globally. Notably, such phase changes can be transient; for example, during the peptides crossing the membrane in order to equilibrate concentration gradients between the outer and the inner leaflet of the membrane [66] (cf. above). Transient openings also occur because the peptides diffuse laterally, thereby stochastic fluctuations in the local peptide-to-lipid ratio occur locally [72]. Furthermore, phase diagrams are a convenient way to represent different modes of interactions between the peptides and lipids where the supramolecular morphologies—such as bilayer, wormholes, tubular structures, bicelle, micelle, or hexagonal phases—depend on the peptide-to-lipid ratio, the detailed membrane composition, temperature, hydration, salt, pH, etc. [58]. For example, for a number of peptides, in-plane or transmembrane topologies have been observed depending on pH, hydration, peptide concentration, and lipid composition [42,124,125,126]. In a recent investigation using dual polarization interferometry, surface plasmon resonance and atomic force microscopy, membrane disordering, associated mass, and structural changes were followed in real-time revealing a number of intermediate states including the lysis and recovery of membranes in the presence of magainin 2 [127]. Furthermore, changes in line tension have been suggested to be a common mechanism for a wide variety of AMPs, observations that are in good agreement with the ideas of the SMART model [127,128].

Within this model at low peptide concentrations, the bilayer structure is maintained, where only transient and local openings may appear. At higher peptide concentrations, an increasing strain on the membrane packing results in openings [65] and macroscopic phase transitions of the peptide-lipid assembly [115]. Thereby, the phase boundaries represent threshold concentrations where the peptides change their level of activities. Thus, in-planar helix orientations agree with both the disruption of the bilayer integrity at higher peptide-to-lipid ratios, as suggested by the ‘carpet model’ [119], and the stochastic and transient rupture and closure of the membrane as revealed by electrophysiological recordings when the peptide concentration is low [59,60,61].

In this context, it is noteworthy that magainins, carrying several positive charges, have been shown to interact better with membranes carrying a negative surface charge from anionic lipids or lipopolysaccharides. Indeed, such preferential association forms part of the explanation why these peptides kill bacteria or tumor cells, which expose negative charges to the outside, and are non-toxic to healthy eukaryotic cells (which are charge-neutral at their outer membrane leaflet) [129,130,131,132,133]. This preferential association can be dissected into an attractive electrostatic interaction that causes a number of orders of magnitude of increase in local surface concentration along the negatively charged surface, and a hydrophobic insertion characterized by partitioning coefficients that are of similar order of magnitude for all membranes investigated (around 1000 M^−1^) [131,134]. Changes in electrostatic interactions upon membrane association of multicationic antimicrobial peptides have also been suggested to result in the release of peripheral membrane proteins, thereby exerting antimicrobial activities [135].

Beyond increases in the apparent association constants through electrostatic interactions, modulation of pore-forming and antimicrobial activities arises from the effect of anionic lipids on the helical penetration depth and/or the topological equilibrium of the cationic peptides [136]. In addition, the formation of domains enriched in cationic peptides and acidic phospholipids has been postulated from ^2^H solid-state NMR experiments using selectively deuterated lipids [137,138]. Electrostatics also play an important role for the interactions between peptides in lipid-mediated mesophase-like arrangements along the membrane surface (Figure 1E) [74], an observation that requires further investigation.

According to the SMART model, other cationic amphipathic molecules have the potential to also exhibit antibacterial activities. Designed antimicrobial compounds should accumulate at the surface of negatively charged bacteria and intercalate into their membranes at the level of the phospholipid headgroups due to hydrophobic interactions. Interactions with healthy eukaryotic cells and toxicity should be avoided by tuning the composition to an overall moderate hydrophobicity. Furthermore, the compounds should not insert too deeply into the lipid bilayer or span the lipid membranes, but they should exhibit interfacial partitioning. Indeed, compounds with such features have been designed and exhibit potent antimicrobials activities. These include short peptide sequences [139,140,141,142,143,144,145], peptide mimetics [29,30,31,32,33,34,35,36], amphipathic polymers [37], or organic molecules encompassing an aromatic ring system, a hydrophobic chain, and cationic functional groups [27].

## 2. Synergistic Enhancement of the Activities of Antimicrobial Peptides

The efficiency of antimicrobial compounds can sometimes be potentiated by applying them in combination [50,146]. For example, mixtures of peptides with conventional antibiotics [37,145,147,148,149,150] or ions [151] show synergistic enhancement. Whereas this enhancement can in some instances be explained by one compound paving the way for the active antimicrobial ingredient [152], other combinations, of e.g., dermaseptins or of bacteriocins, seem to interact more specifically to exhibit synergistic activity [50,153]. Synergistic interactions involving magainin 2 have been detected with PGLa [154], or with the cyclic beta-sheet peptide tachyplesin I [155].

The combination of magainin 2 and PGLa does not only show increased killing efficiency of bacteria, but also a more efficient release of calcein from liposomes made from phospholipid bilayers [75,156,157]. It is interesting to note that these peptides are naturally stored as a cocktail in the skin of *Xenopus laevis* frogs. Thus, it seems that the naturally active synergism has been initially destroyed by the standard analytical processes which involve separating the peptides from the complex mixture and investigating each of them individually. In an early investigation on synergism, Masuzaki et al. suggested that the pore formation rate of magainin is slower, but the pores are more stable than those of PGLa [157]. In the mixture, synergism is a consequence of combining fast pore formation and moderate stability. More recent work by Heerklotz and co-workers suggest that synergistic vesicle leakage is a result of optimizing the size of the pores and their distribution among the liposomes [158]. They should be large enough, but at the same time sufficient in number to cause dye release from all vesicles in the suspension. The propositions by both laboratories are related to the heterogeneity of the peptide distribution in the membranes, and thereby related to the size of the vesicles or of bacterial cells [158]. An additional ingredient to be considered is the possibility that one factor of the synergistic mixture solubilizes the second one, thus increasing its availability [158].

In equimolar peptide mixtures, PGLa and magainin have both been found to exhibit an alignment parallel to the membrane surface, provided that the membranes carry at least one unsaturation per phospholipid [95,159,160], such as in *E. coli* lipid extracts [161] (Figure 1E–G). This helix topology resembles those when magainin or PGLa are investigated individually by solid-state NMR spectroscopy [38,95]. Contrasting data are obtained from lipids all carrying only saturated fatty acyl chains where magainin remains oriented along the membrane surface, but PGLa, which is present in the same mixtures, flips into transmembrane alignments [95,159]. Notably, in DMPC and in the presence of PGLa, a 30° deviation from perfectly in-planar topology has also been observed for magainin 2 [162]. Because fully saturated lipid mixtures do not represent the physiological membrane composition well, it seems reasonable to assume an in-planar membrane topology for both peptides (Figure 1E–G), and to use the context of the SMART model when mechanistic explanations for the synergistic antibacterial activities are elaborated [43].

In order to develop a structural model of a supposed synergistic complex, experiments were designed to delineate possible interactions sites between magainin 2 and PGLa. For example, modified peptide sequences have been investigated. Early on, during dye release experiments from egg PC/PG (1:1) liposomes, the F16W and E19Q mutants of magainin 2 were shown to exhibit reduced synergistic activity, whereas the F5W mutation did not exhibit any effect [157]. It should be noted however, that a quantitative comparison of synergism is hampered by variations that are observed when different bacterial strains are compared to each other or to model membranes [75,163,164,165]. Importantly in a recent investigation Leber et al. showed that the synergism of calcein release activities from liposomes is not only a function of the absolute peptide concentration, but also of the membrane intrinsic curvature and thus lipid composition [75]. Synergism is most pronounced for membranes with high negative intrinsic curvature involving POPE lipids, and only apparent at peptide concentrations ≥ 0.4 µM [75]. The synergistic factors were much reduced and even abolished for calcein release experiments from POPC/POPG 3/1 where the peptides alone exhibit a high activity [75]. In this context, it is interesting to note that in recent investigations, the antimicrobial activity of magainin and PGLa derivatives leveled out at minimum inhibitory concentrations (MICs) of about 1 µM [161,77]. Therefore, when only the synergistic factor is considered smaller values are obtained for peptides that exhibit already high antimicrobial activity when investigated alone [161]. Similarly, when an α-helical sequence was modified by the insertion of prolines, the synergy with conventional antibiotics increased as the antibacterial effectiveness of the peptides decreased [166].

An extensive mutagenesis study showed that the synergistic activity is abolished successively when removing/inverting the negative charges at E19 and the carboxy terminus of magainin 2 [164]. As for PGLa the positively charged K15 and K19 sites had a favorable effect on the synergistic enhancement [164]. Whereas making the hydrophobic face more hydrophobic increased the antimicrobial efficiency of magainin 2, it had no effect on synergism [165]. Recently, the PGLa residues G7, G11, and L18 have been found to be important for the synergistic enhancement of activities between the two peptides [164]. Although it is interesting that glycines 7 and 11 form a GxxxG motif, which has been shown to drive dimerization of transmembrane helical sequences in highly apolar environments, the PGLa helix is localized at the interface rather than the hydrophobic interior of the membrane [95,161]. Furthermore, there is no GxxxG motif on magainin 2 which could serve as a counterpart for the formation of a magainin-PGLa heterodimer (Table 1). Therefore, to better understand the role of the two glycines and of L18 in promoting synergistic activities, further structural investigations are required.

Coarse-grain MD simulations over several microseconds encompassing 24 peptides (PGLa and magainin) and 512 dilauroylphosphatidylcholine (DLPC; C12:0) lipids were also performed [167]. These simulations confirmed a tilting and deeper penetration of PGLa into the membrane, without adopting a transmembrane orientation, whereas magainin stays on the bilayer surface. The simulations reveal a clustering of the peptides by electrostatic interactions, concomitant with a parallel alignment of the two helices, albeit without the explicit formation of pores [167]. An all-atom MD simulation of the peptide mixture in DMPC and DMPG membranes, starting from transmembrane tetramers was performed. The simulations suggest antiparallel helix arrangements in the 1:1 heterotetramer with stronger interactions in the heterodimer than in the homodimer. Plausible interactions could occur between the S8 and E19 residues of magainin and K12 and K19 of PGLa [108]. Though in the mixture of both peptides the tilt angle of PGLa is reduced when compared to PGLa alone, a large variety of helix alignments relative to the normal membrane still persists [108].

Fluorescence spectra were used to derive constants for the membrane association of magainin and its interactions with PGLa in its membrane. Favorable PGLa-magainin interaction energies were obtained when investigated in egg-PG membranes, where the exact value depends on the assumed numbers of peptides involved in the process [157]. Energies for homo- and heterodimer formation based on the midpoints of the concentration-dependent transition of PGLa from an in-planar to a transmembrane alignment were extracted in a later investigation [168]. Notably, this transition only occurs in fully saturated membranes, but not in the presence of lipid unsaturation, as they occur in biological membranes. One should also be aware that these quantities are associated with a specifically chosen interaction model (cf. reference [157]). Furthermore, they represent a multitude of interaction terms that change during the topological transition; thus the energies involved include the transfer of residues in between polar and non-polar membrane environments, and energies associated with disordering the lipids or local changes in membrane phase, direct interactions between the peptide and the lipid as well as between peptides [104,125]. In this context, it should be noted that fluorescence resonance energy transfer (FRET) experiments did not reveal a strong interaction between magainin 2 and PGLa when associated to POPE/POPG 3/1 or POPC/POPS 3/1 membranes [77].

**Figure 2:**
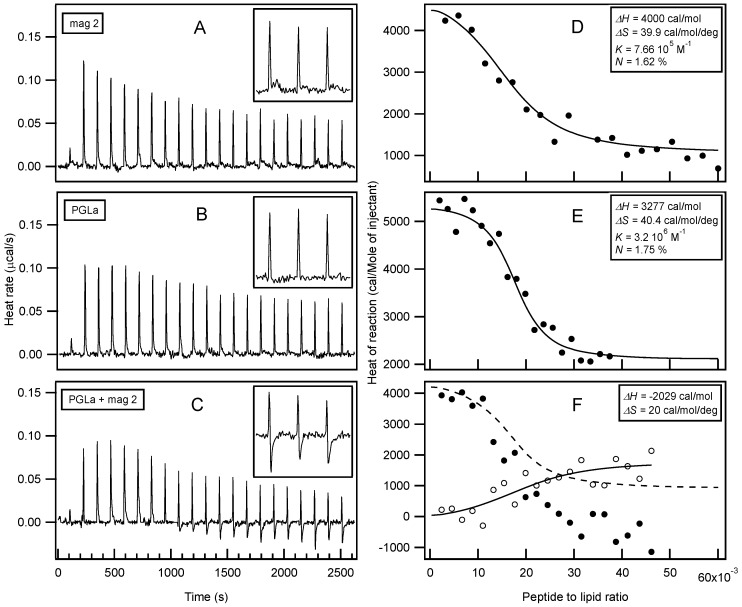
Isothermal Titration Calorimetry measurements of large unilamellar vesicles (LUVs) made of palmitoyl-oleoyl-phosphatidylethanolamine/ palmitoyl-oleoyl-phosphatidylglycerol POPE/POPG (3/1) at 440 µM total lipid concentration, into which solutions of 85 µM magainin 2 (**A**), 140 µM PGLa (**B**), and the equimolar mixture of PGLa and magainin 2 at 105 µM total peptide concentration (**C**) have been injected successively. Buffer: 10 mM Tris-HCl, pH 7, 100 mM NaCl. The inserts show close-ups of the regions *t* > 2215 s. The fittings of the data with a single binding site model are displayed as solid lines in panels **D**–**F**. The open circles in panel **F** show the difference of the heat of reactions when comparing the mixture of peptides with the sums obtained from the magainin 2 and PGLa titrations (i.e., half the intensities of the fitted curves shown in panels **D** and **E**, dashed line). The values of the enthalpies (Δ*H*), entropies (Δ*S*), binding constant (*K*), and apparent stoichiometry (*N*) are reported in the corresponding graphs.

Isothermal Titration Calorimetry (ITC) has already provided valuable insights into the thermodynamics or membrane association of magainin 2 and PGLa [56,131,134,169,170,171]. In order to further explore possible interactions with membranes, large unilamellar vesicles (LUVs) made of POPE/POPG 3/1 at pH 7 were prepared as a model system for bacterial membranes. In this context, the interactions of both peptides individually and as a mixture were investigated (Figure 2). Interestingly, only endothermic enthalpies (Δ*H*) were observed when each peptide was titrated into the lipid suspension individually (Figure 2A,B) while the peptide mixture revealed a considerably more complex time trace of reaction enthalpies (Figure 2C), suggesting additional modes of interaction. Additional exothermic enthalpies are observed for peptide to lipid molar ratios > 1.5%, i.e. for times of injections *t* > 1000 s. When compared to previous investigations with 30 nm small unilamellar vesicles (SUVs) [131,134,169,170], the reaction enthalpies of magainin 2 and PGLa with 100 nm LUVs, also used here, are relatively small [171]. A quantitative analysis of the ITC traces (Figure 2D,E) reveals enthalpies of 3–4 kcal/mol for PGLa and magainin, respectively, entropies of 40 cal·mol^−1^·K^−1^, and apparent membrane association constants in the 10^6^ M^−1^ range (apparent stoichiometry P/L ≈ 1.7 mol %). Although different experimental conditions have been chosen for the experiments shown in Figure 2 when compared to previous investigations, a closely related stoichiometry becomes apparent [134]. By comparing the enthalpy produced from titration of the mixed peptide solution with the combined data from the individual injections, a Δ*H* in the range of −2 kcal/mol remains for additional processes in the peptide mixture (Figure 2F).

**Figure 3:**
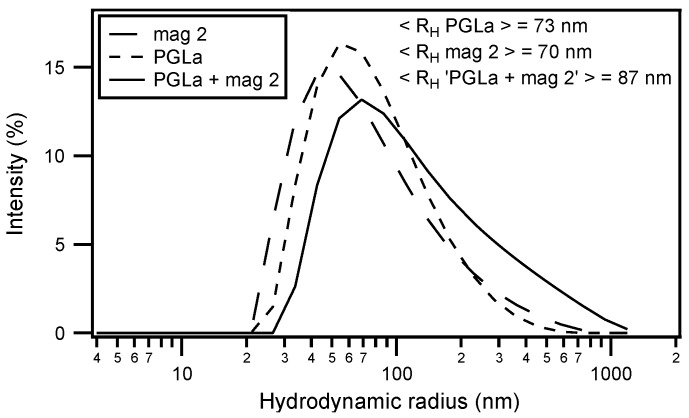
Dynamic light s cattering (DLS) size measurement of LUVs made of 440 µM POPE/POPG (3/1) and incubated with magainin 2 (long dashed line), PGLa (short dashed line) or an equimolar mixture of PGLa and magainin 2 (continuous line) in 10 mM Tris-HCl buffer pH 7. The vesicles were made by mechanical extrusion through a 100 nm pore filter and the peptide to lipid ratio was kept constant at P/L = 3.6% for all tree measurements (this corresponds to the same condition for peptide injection at *t* ≈ 2030 s in Figure 2C). The averaged hydrodynamic radius < R_H_ > is indicated in the upper right of the graphs.

Circular dichroism (CD) analysis (not shown) combined with Dynamic light scattering (DLS) measurements were performed under the same conditions (Figure 3). Both peptides adopt largely α-helical conformations while they interact with the membrane. At the same time, large supramolecular structures form, suggesting the flocculation of the vesicles in the simultaneous presence of both peptides, similar to observations made with a designed model antimicrobial peptide [76,172]. Previously, a reduction in bilayer repeat distance of mechanically oriented membranes in the presence of magainin and magainin/PGLa, but not PGLa alone, has been observed [103]. It seems possible that these previous observations are related to the interbilayer interactions observed in our ITC and DLS experiments (Figure 2 and Figure 3). Figure 1F schematically illustrates the possible role of peptide–peptide interactions during such processes. Membrane pore formation by the AMP mastoparan-X and micellation at much higher P/L ratio was previously reported from ITC data [173].

In order to further explore possible interactions between membrane-associated PGLa and magainin 2, cross linking experiments have been performed with peptides carrying a GGC extensions [174]. This work shows that when added to egg PC/PG(1/1) lipid membranes, parallel dimers preferentially form [174]. Based on this data, covalent dimers linked through C-terminal GGC extensions were prepared, and all the (PGLa-GGC)_2_ and (magainin-GGC)_2_ homodimers, as well as the magainin-GGC/PGLa-GGC heterodimer were more active in calcein release from POPE/POPG 3/1 liposomes, than with the same amount of unmodified peptides in a mixture [75]. However, when investigated in POPC/cholesterol 3/1 mixtures, quite different results were obtained because only the PGLa-homodimer and the PGLa-magainin heterodimer, but not the individual peptides or their mixture, showed significant release activities [75]. Thus, the increased activities of the dimers seem not to be related to a particular structure formed by the combination of PGLa and magainin 2, but rather they reflect the increased membrane-perturbing properties of larger peptide aggregates [175,176,177]. Notably, the comparison of dimer and monomer antibacterial activities of wild type magainin and PGLa and their derivatives are complicated by the fact that they are already increased by the GGC extensions [161,163].

There are only few biophysical investigations that elucidate the mechanisms of the synergism observed for membrane-associated amphipathic peptides. Nevertheless, our view has already moved from models of heterooligomeric transmembrane bundles to helices that somehow play together when being oriented at the membrane surface. Thereby the situation resembles early research on cationic amphipathic peptides which had been found to reside at the membrane interface rather than forming transmembrane helical bundles [178,179]. From there the field has developed [46,58,180,181] and after years of research still bears surprises [74]. It can be expected that our views on synergism will evolve in a related manner [75,95,158,159].

Recently it has been shown that synergism is most pronounced when the peptides alone have not reached their optimum [75,161]. In good agreement with the SMART model [43], the peptide sequence and the membrane lipid composition are both important elements of synergy [75,161]. Thus, synergistic enhancements of calcein release only becomes apparent in membranes with intrinsic negative curvature and negative surface charge [75]. It is suggested that PGLa preconditions the more densely packed POPE membranes by softening up its interface, thus magainin, being more amphipathic, can penetrate deeper and be more active (Figure 1E) [75]. Furthermore, it is of interest for our understanding of the mechanism of action of antimicrobial peptides *per se* and the synergism they develop in their membrane associated state, that fluorescence quenching experiments are indicative of mesophase arrangements of both peptides along the membrane interface and a more densely packed supramolecular arrangement when both peptides are present in equimolar quantities [74,77] (Figure 1D,G).

## Figures and Tables

**Table 1:** Sequences of peptides discussed in this paper. The one-letter code is used for peptides made from conventional amino acids only. The alamethicin sequence is given by the three-letter code with the following non-standard residues: Aib: α-aminoisobutyric acid, Phl: L-phenylalaninol, Ac- for acetyl- and -NH_2_ for the carboxamide terminus, respectively.

magainin 2	GIGKF LHSAK KFGKA FVGEI MNS
PGLa	GMASK AGAIA GKIAK VALKA L-NH_2_
cecropin A	KWKLF KKIEK VGQNI RDGII KAGPA VAVVG QATQI AK-NH_2_
LL37	LLGDF FRKSK EKIGK EFKRI VQRIK DFLRN LVPRT ES
melittin	GIGAV LKVLT TGLPA LISWI KRKRQ Q-NH_2_
LAH4	KKALL ALALH HLAHL ALHLA LALKK A-NH_2_
alamethicin (F50/7)	Ac-Aib-Pro-Aib-Ala-Aib-Aib-Gln-Aib-Val-Aib-Gly-Leu-Aib-Pro-Val-Aib-Aib-Gln-Gln-Phl

## Abbreviations

Aib	α-aminobutyric acid
AMP	antimicrobial peptide
CD	circular dichroism
DLPC	1, 2-lauroyl-*sn*-glycero-3-phosphocholine
DLS	dynamic light scattering
DMPC	1, 2-dimyristoyl-*sn*-glycero-3-phosphocholine
DMPG	1, 2-dimyristoyl-*sn*-glycero-3-phospho-(1′-*rac*-glycerol)
DOPC	1, 2-dioleoyl-*sn*-glycero-3-phosphocholine
DOPG	1, 2-dioleoyl-*sn*-glycero-3-phospho-(1′-*rac*-glycerol)
GUV	giant unilamellar vesicle
IP	in-plane
ITC	isothermal titration calorimetry
LUV	large unilamellar vesicle
MD	molecular dynamics
NMR	nuclear magnetic resonance
PC	phosphatidylcholine
PE	phosphatidylethanolamine
PG	phosphatidylglycerol
POPC	1-palmitoyl-2-oleoyl-*sn*-glycero-3-phosphocholine
POPE	1-palmitoyl-2-oleoyl-*sn*-glycero-3-phosphoethanolamine
POPG	1-palmitoyl-2-oleoyl -*sn*-glycero-3- phospho-(1′-*rac*-glycerol)
POPS	1-palmitoyl-2-oleoyl-*sn*-glycero-3-phosphoserine
SMART	Soft Membranes Adapt and Respond, also Transiently
TM	transmembrane
